# To Be or Not to Be a Chechen? The Second Generation of Chechens in Europe and Their Choices of Identity

**DOI:** 10.3389/fsoc.2021.631961

**Published:** 2021-03-31

**Authors:** Marat Iliyasov

**Affiliations:** School of International Relations, University of St Andrews, St Andrews, United Kingdom

**Keywords:** Chechen, migrant, second-generation, Europe, collective identity, original identity

## Abstract

Approximately a quarter of Chechnya's population left the republic due to the Russo-Chechen wars and the brutality of the regime established after them. Many of the Chechen migrants settled in Europe where cultural, religious, and social differences compelled them to go through the daunting process of identity negotiation. Although most of the first-generation Chechen migrants managed to preserve their original identity, this was not always the case for their children. This article aims to identify the factors that determine the identity preferences of second-generation Chechens in Europe. The paper presents three cases which illustrate very different outcomes of the identity formation and negotiation processes. This ethnographic study concludes that home education impacted the identity choices of the migrants' children the most.

## Introduction

In contrast to the first Russo-Chechen war (1994–1996), the second one (1999–2009) resulted in a large-scale out-migration of Chechens. Many emigrants headed to Europe. The first large wave of Chechen refugees reached the European Union in the year 2000. Since then, the flow of migrants from Chechnya to the EU persisted, despite decreases in numbers of migrants. As a result, during the last two decades, the population of Chechens in Europe, according to very cautious estimates, rose to ~200,000 people (Williams, 2016: 229; Kirilenko, 2017; Laruelle, 2017).

Like other migrants, Chechens went through the identity negotiation process, which to some extent transformed their “original”^1^ identity. The loss of original identity is more obvious amongst the children of these migrants, however. Chechen social media often circulates stories about one or another compatriot who is engaged in an enterprise that “does not suit Chechen behavior.” These stories about those who have “betrayed” their ethnic or religious identities produce cries of outrage among Chechen users of the Internet. For instance, a video from July 2020, which presented a story of a “former Chechen Muslim” who was gradually turning toward Christianity through the revelation of Jesus, was arduously discussed in various online forums. The interview with this young Chechen man, who was stigmatized by his father for being disobedient, collected 251,189 views by October 7, 2020^2^. Another story, which presents a young Chechen woman who became a member of the German Bundestag in 2020, is also among the notorious ones. It maintains that this young lady is not a Chechen anymore because she does not follow Chechen norms of behavior. Instead, she “wears trousers, eats pork, drinks beer, and does not observe Chechen etiquette^3^.” The video, which is named “Ona ne chechenka” (“She is not a Chechen” - Russian), was posted to YouTube on February 2020 and collected 64,766 views by the same date (October 7, 2020). This is not to say that all second-generation Chechens in Europe fail to preserve their parents' identity and do not behave as it prescribes. As the three cases of the Chechen teenage males analyzed in this article demonstrate, some of the second-generation Chechens in Europe succeed in it. The very different outcomes of identity formation and negotiation processes inform the question of this research: What drives second-generation Chechens in Europe to preserve or to abandon the identity of their parents? To answer this question, this paper identifies the crucial factors in the process of collective identity formation of young Chechens in Europe.

Little has been written on identity of Chechen migrants' in Europe. Just a few studies by Molodikova (2019), Sipos (2020), and Szczepanikova (2012, 2015) explore different aspects of ethno-cultural collective identity of Chechen refugees. The young age of Chechen community in Europe (approximately 20 years) explains why only one study by Kość-ryzko (2015) targets the question of identity formation of Chechen migrants' children.

This study is an attempt to encourage further research of Chechen migrants' community that provides a perfect laboratory for exploration of refugees' children identity formation and negotiation processes. More important, it elaborates on the Kość-ryzko (2015) identified socio-economic and cultural factors that influence the outcome of these processes. The article builds its theoretical framework using constructivist approach to collective identity formation (Tajfel and Turner, 1986; Eriksen, 1993) with the focus on social milieu migrants' families live in (Berry, 2001; Phalet and Schönpflug, 2001; Crocetti et al., 2011). The theoretical contribution of this paper is limited by confirming the existing insights on adolescence identity formation and negotiation processes (Crocetti et al., 2011; Guzder, 2011; McGregor et al., 2015, 2016) through the analyzed cases. The analysisincludes the views of the first-generation Chechen migrants, the explanations of the youngsters themselves, and the author's personal observations.

The article proceeds as follows. First, it presents the methodological consideration employed and the ways in which the data was collected. Then it examines leading published studies on identity formation in migrant communities (Berry, 2001; Crocetti et al., 2011; Rabiau, 2019), which serve as the theoretical foundation for this paper. After that, the study proceeds with the case. After outlining the original identity of the first-generation Chechen migrants in Europe, the paper explores the identity choices of their children. The article concludes by bringing to the fore some of the factors that, according to this research, played a crucial role in the identity formation of young Chechens in Europe.

## Approaching Migrants' Identity Methodologically

To begin with, it should be noted that this ethnographic study is of a qualitative nature. The qualitative approach of this study is determined by its focus on collective identity. Even though there have been attempts to quantify research on collective identity (Paul and Fischer, 1980; Parham and Helms, 1981; Phinney and Chavira, 1992; Phinney, 2000), there efforts have not always proven to be successful due to an obvious limitation – identity is difficult to measure (Albert, 2014), categorize, or generalize. Moreover, being of a positivistic nature, such attempts usually ignore the reasons behind people's choices and qualitative research, on contrary, focuses on them (Maleševic, 2003). By choosing a qualitative research methodology, this study tries to avoid such determinism and give space to informants in explaining their self-perception and presenting their consideration regarding personal identity choices.

Having acknowledged the evolving nature of identity (Eriksen, 1993; Mach, 1993), for the sake of exploration, this paper follows a suggestion of Abdelal et al. (2009, 28) and attaches identity to a certain point or period of time for the sake of describing it. Thus, describing the first-generation Chechen migrants' original identity the article refers to their identity formed before their departure from Chechnya. The detailed description of this identity is available in the scholarly literature (Jaimoukha, 2004; Sokirianskaia, 2005; Gammer, 2006) that the article relies on. In addition to this, this paper refers to the author's research of Chechen migrants conducted in Europe between 2014 and 2017. The data collected during the later fieldwork (2017–2020) focuses on the identity of second-generation Chechens in Europe (attached to the time of research). This dual focus aallows for an uncovering of the differences between the identities of the first and second generations of the Chechens in Europe. Furthermore, it helps to identify the factors that determine the formation of migrants' children identity.

The data on identity formation of second-generation Chechens in Europe was collected during two sets of fieldwork in 2017 and 2020 with in two different European countries. In both countries, the author was invited to be the guest of a Chechen family for 5–6 weeks. During this time of fieldwork, the author closely observed seven Chechen families in total. This study presents only three cases because these samples best illustrate the different outcomes of the identity formation/negotiation process among second-generation Chechens in Europe. In other words, the cases were chosen to present the most and the least successful transmission of original identity out of studied.

To collect data, I combined in-depth interview and participant observation methods. The data collection mostly took the form of extensive conversations and observation of families informed by behavioral models. On average, I have spent between 10 and 12 h with the members of the observed families conversing about the topic of my interest. Naturally, I developed a closer connection with the two families I stayed with. These cases enriched my study by enabling me to produce deeper observation and to witness the occasional comments made by the participants during their casual daily encounters. In accordance with research ethics, the informants were duly informed of my intention to research and publish the results of the investigation. The participants were also ensured that this article would observe their rights and maintain their anonymity by replacing their real names and by avoiding any mentioning of their whereabouts. These precautions are taken as the comparatively small size of Chechen communities in Europe would make it easy to identify my informants.

The first-generation migrants of all the studied families (except one for extended family) were approximately the same age (between 45 and 55 years old), but from different professional and educational backgrounds. All the children under observation were males in their late teenage years. This gender choice was dictated by the strict Chechen traditions that do not allow private communication of a non-relative male and an unmarried female^4^. My relationship with the observed teenagers developed through my participation in their activities, which I was also curious to learn about. In most instances they were happy about my being present and were eager to talk about their interests, plans, and views. They were aware that I was studying them, yet they did not object to some information about their personal lives becoming public. I could see that they were even flattered by this idea. The total amount of time that I have spent in direct contact with the studied youth could also amount to around 10–12 h. However, I did not always have a chance to engage in conversation with them during the time I spent with them. This was another reason for favoring the method of participant observation. In general, my fieldwork allowed me to make relevant observations and collect explanations regarding identity formation from the point of view of both Chechen generations in Europe.

## Migrants' Identity Formation in Theory

As mentioned in the previous section, identity is a fluid phenomenon, which develops constantly as a reaction to habitat. This characteristic of identity has been emphasized in many studies, which have also described identity as complex, continuously evolving, multi-layered, and situational (Tajfel and Turner, 1986; Mach, 1993; Baumeister, 1995; Guzder, 2011). The fluidity and continuous evolution of identity explain why even a slight alteration in one's social milieu might initiate a process of identity modification (Kinnvall, 2004). It is not surprising that migrants and their families are particularly exposed, vulnerable, and liable to identity changes. These changes occur despite a powerful drive to maintain a sense of self-identity (Sigel, 1989, 459). Indeed, it is hardly possible for a migrant to maintain his/her original identity, as outside forces are usually strong enough to encourage a newcomer to negotiate his/her identity vis-à-vis the cultural practices of the host society (Triandis, 2001; Burke, 2006). The process of negotiation, which is influenced or driven by social interaction (see Berry, 2001; Phalet and Schönpflug, 2001), is not the same for migrants of the first-generation and their children (Eriksen, 1993).

Unlike their parents, who lived in their original society, the children of migrants have a different initial ground for forming and negotiating their identity (see Table 1). Three main components constitute their identity construction process (Crocetti et al., 2011; McGregor et al., 2015, 2016; Rabiau, 2019). First, they are being raised in migrant families, whose members might try to keep them within the frame of original ethnic identity or let them choose their own identity freely. Second, they are exposed to the influence of the host society to a larger extent than their parents (Berry, 2001; Phalet and Schönpflug, 2001). Third, they have the example of other migrant communities within the host society (Verkuyten and Kinket, 2000; Triandafyllidou, 2009).

**Table 1:** The sources of migrants' identity.

**Sources**	**Identity of migrants**	**Identity of migrants' children**
Primary – the source of initial information that influences identity formation process.	Family (homeland, [**primary source**])	Family (out of homeland, [**primary source**])
Secondary – the main (out of family) source of information that influences the identity construction process.	Original society [**main source**]	—
Tertiary – the alternative sources (sometimes of little importance) of information that influences the identity formation process.	Hosting society [**secondary source**]	Hosting society [**main source/secondary source**]
Main – the source that provides vital information for identity construction.	Other communities (within hosting society [**tertiary source**])	Other communities (within hosting society [**tertiary source**])

*Table is comprised by the author basing on the works of Berry (2001), Phalet and Schönpflug (2001), and Triandis (2001)*.

The first component pertains to the communication dynamics within a family, which, according to McGregor et al. (2015, 2016) determine the extent to which “culture and meaning making are transmitted and nurtured.” Indeed, some migrant families have a very strong emotional attachment to their home country or culture. Such families might try to preserve their ethnic identity against all odds serving as “an anchor for identity in exile” (Rousseau et al., 2001). Other migrant families may choose the option to integrate or become assimilated. They consider the host country as their new home and try to rebuild their lives and to re-construct or adjust their ethnic identity with this perspective. Based on their personal characteristics (Crocetti et al., 2011), intergenerational conflict (Idema and Phalet, 2007), and the attitude of the host society (Verkuyten and Kinket, 2000; Triandis, 2001; Kosic et al., 2005), migrants' children may form a new identity which falls somewhere along the spectrum and in between the two contrasting options. Even in traditional families, migrants' children are generally inclined to move further away from the original identity that their parents exhibit due to the following reasons. First, migrants' children are already familiar with the identity negotiation process as they have witnessed their parents going through this very process. This makes the migrants' children aware that the identity that they receive from their parents is already modified. This encourages them to search for their own identity. Second, the uninviting reactions of migrants' children may provoke an attempt oftheir parents to sell them a negotiated identity passed as original. Third, the element of originality only deteriorates as migrants' children construct their own identities themselves. In some cases, however, the children of migrants may try to restore this element of originality.

The second factor that plays a significant role in constructing the identity of the migrants' children is exposure to a host society (Sleijpen et al., 2016; Rabiau, 2019). Children's engagement with the identity of a host society through schooling, friends, and participation in cultural events can reinforce or undermine the initial impetus regarding their identity choices received from the family. Either way, daily observation of the cultural practices of a host society can encourage migrants' children to tolerate even models of behavior that would be unthinkable or unacceptable for them had they been raised in the original society. Furthermore, their knowledge of the culture, traditions, and history of the original society grows weaker as migrants' children become more immersed in the host society and gain more knowledge of local traditions (Druckman, 1994). Migrants' children also might not share the political convictions or traumatic experience (Booth, 1999) that kept the collective identity of their parents salient. As a result of this cultural setting, in many cases, children adopt not only a tolerant view, but also some social practices, and, in many cases, the language of the host society as their first or even preferred choice.

The assimilation of migrants' children occurs even faster in places with a low concentration of members of their original ethnic group, which is not so unusual in the territorially large countries with small populations or when a migrant community is comparatively small. In such cases, as Luo and Wiseman (2000) found the prominence of original identity decreases dramatically and the reduction in ethnic behavior becomes even more observable (Lee, 1999). Modern technology does not help much to preserve the original identity either, even if first-generation migrants encourage their children to develop an online link with the homeland (Szczepanikova, 2012). Internet communication rarely builds a strong enough emotional connection to replace emotional attachment to physically available friends. Therefore, unlike the older generation, which already formed this emotional bond (with friends and relatives in the original society) and attempted to maintain it, youngsters use the Internet to communicate with those who are in geographical or physical proximity, unless they already have an emotional bond established.

The influence of other migrant communities, which is the last component to be considered in this paper, is tightly linked to the experience of migrants' children in the host society. The attitude of the host society in a broader societal context, as some scholars confirm, is especially critical in forming the identity of adolescents (Hughes and Johnson, 2001; McBride Murry et al., 2001) but also influences adult migrants in their choices. In general, displayed indifference, negativity, or prejudices against the migrant populations antagonize the migrants' communities and host societies (Adams and Marshall, 1996; Bosma and Kunnen, 2001). The host society can even become a significant “other” against whom the migrants' identity is constructed (Ackroyd and Pilkington, 1999; De Castro, 2004; Devine and Kelly, 2006; Ryan, 2010). In such cases, migrants can choose to communicate or associate with other migrant communities rather than the original (non-migrant) members of the host society (Verkuyten and Kinket, 2000; Triandafyllidou, 2009). Moreover, negativity toward or from the host society received or expressed by the first-generation migrants is easily transmitted to their children and vice versa, which also determines migrants' communication choices. On the contrary, a lack of hostility, as Kibria (2002) found, encourages integration into the culture of the majority. In other words, different attitudes toward migrants can result in the desire to accept or reject the culture of the host society and, consequently, to merge or not with the other communities of migrants.

In sum, in comparison to their parents, the children of migrants are more vulnerable to losing their original identity to the host society. In addition to the general physical adaptability of children to new situations, there are three more reasons that determine this. First, they are exposed to the host society to a greater degree than their parents are. Second, they receive an already negotiated original identity from their families. Third, they usually have limited communication with their society of origin due to geographic distance and weaker emotional attachment to their homeland in comparison to their parents. This is not to say that the children of migrants always lose the original identity which their parents formed. Depending on his or her personality (see Crocetti et al., 2008), a child might even choose to return to his or her homeland.

## The Original Identity of Chechen Migrants

To identify the difference between the identity of first-generation Chechen migrants in Europe and their children, it is necessary to outline the original identity of the former. However, the possibility of this exercise is highly limited by the fluid nature of identity, multi-layered structure of society, and the complex individual process of identity formation (Eriksen, 1993; Mach, 1993), which may produce a very different outcome in each individual case. In other words, it is rather difficult to give a precise answer to the question of what constitutes the original identity of the first-generation Chechen migrants. Furthermore, it must be kept in mind that constantly evolving nature of identity (Tajfel and Turner, 1986; Kinnvall, 2004) does not comply with drawing borders nor is it congruent with building rigid frames of group identity. Being a highly situational phenomenon (Crocetti et al., 2011), collective identity allows for a certain flexibility in the interpretation of which behavior is accepted by an ethnic group and which is not. Therefore, the red lines that a Chechen should not cross to maintain his/her membership in the group can be pushed further away or kept firmly where they are depending on one's personal view. This flexibility is also related to the fact that there is no actual authority that can decide or validate a person's membership in an ethnic group. Besides the briefly observed issues of sketching an original identity, there is still a need to have a point of departure that would allow the paper to compare and identify the changes underwent by the second-generation Chechens in Europe. To reinforce this point, it is necessary to pin down the dominant characteristics of the 35–50-year-old Chechens who constitute most of the first-generation Chechen migrants in Europe. The identification of these characteristics is possible through analysis of the social milieu and the factors that influenced the formation of collective identity of this age cohort. To complete the picture, it is also necessary to place the identified characteristics within the frame of Chechen culture and religion.

To start with, first-generation Chechen migrants in Europe were mostly raised in Chechnya. The region, though culturally diverse during Soviet times, mainly consisted of the Chechen majority and, as censuses of 1979 and 1989 suggest, has been steadily growing homogenous^5^. Despite the great influence of Soviet/Russian culture and education, the pre-dominance of the Chechen population ensured a widespread Chechen language and comprehensive knowledge of traditions by the target group. The cultural practice of communal education, in which an older person assumes the role of explaining to a younger person how s/he is supposed to behave, resulted in the successful transmission of ethnic traditions and religious norms. One of my interviewees recalls:

I was raised in the city [Grozny], where most of the population was Russian. My parents were busy with work and they placed me in a boarding school, where I would stay from Monday to Saturday. The weekends I would spend with the family of my father in a small Chechen town, but this was not enough to balance the whole week spent among Russians. Therefore, I did not know many Chechen traditions. One weekend, as usual, I was returning to my father's town. I went off the bus and started walking toward the house, where we lived. On my way, I met an old person and being accustomed to the city life, I passed without greeting him. I still remember the look of surprise on his face when he watched me passing silently. Once I passed him, he addressed me saying: “Good morning, little *k'uonakh* (a knight - Chechen).” I never passed an older person without greeting him or her again in my life. That's how children used to learn traditions in Chechnya when I was young. You knew what to do during the Ramadan from the behavior of others, how to congratulate people with the end of fasting, how to express condolences or how to pray. If not parents, your friends or relatives would teach you (Chechen male, 56 years-old, interviewed in Belgium, March 2017)^6^.

The recollection of this interviewee demonstrates one of the ways by which Chechen society would transfer the models of traditional practices in the 1970–1980s. Besides direct family education, other ways of transmitting the cultural practice would be through living in extended and highly patriarchal families; communal works, which would follow by festivities; weddings; and funerals (Jaimoukha, 2004; Sokirianskaia, 2005; Gammer, 2006). All these practices contributed to the passed down knowledge of traditional Chechen practices.

Furthermore, it is important to mention that the spoken Chechen language would always dominate both the public and private spheres within the Chechen populated rural areas (Tishkov, 2004, 152). The population's level of Chechen language skills could not contend the level of Russian, however. The latter was the official language taught in school and would eventually become leading among the Chechen population, especially in academia. Chechen literature was scarcely published and was thus insufficient in raising the level of the Chechen higher. Nevertheless, the extensive knowledge and wide use of the spoken language, as well as the transmitted models of traditional behavior, taught the cohort under investigation the basics of Chechen cultural practice. As one of my interviewees stated, adhering to the Chechen culture would be impossible without knowledge of the Chechen language.

Of course, language is crucial. How can you teach and explain to your children what is “Yah” (“Rivalry of honor” - Chechen) or “Ghillakh” (“Courtesy” – Chechen), who is a “K'uonakh” (“Knight” - Chechen), and how to be “Oesdan” (“Noble” - Chechen) without knowing Chechen language. There is no translation that transmits the full meaning of these words. Even if you find a way to translate them, it will not be the same. They [these words] can be used and explained only in Chechen language and should be endorsed by Chechen behavior (Chechen male, 47 years-old, interviewed in Belgium, March, 2017)^7^.

This understanding of the importance of the mentioned (and other) words also found in Patackas (1999) anthropological study of the Chechens. He confirms that these (and some other) Chechen words, which he calls the “keywords that unlock the essence of the nation,” are too rich to be translated to other languages by one word. Rather, they are concepts that include and imply certain models of social practice.

The political changes of the 1990s, triggered by the dissolving Soviet Union, brought up issues that were hidden beneath the surface. The traumatic experience of the Chechen deportation and exile of 1944–1957 became an impetus for the nation's political mobilization, declaration of the republic's independence, and consequent resistance to the Russian military invasion of 1994 (Williams, 2000). The victory against Russian forces in 1996 together with the aforementioned factors contributed to the reinforcement of the Chechen collective identity. It can be claimed that observation of religious rites, following cultural norms of etiquette, attention to the dress-code, and wide use of Chechen language prevailed in Chechen society before the onset of the second Russo-Chechen war of 1999–2009. It is not to say that during that time-period the nation was homogenous and united. There were different religious and ethnic groups, which followed their own cultural practices or even opposed Chechen traditional norms (e.g., Salafists).

In sum, the societal factors such as the communal life of extended patriarchal families in rural environments coupled with the political and military perturbations of the 1990s and 2000s were among the main drivers that constructed nationalism-based collective identity of the age cohort (30–50 years old) dominant among the first-generation Chechen migrants in Europe. Cultural etiquette and religious practices, which were taught in Chechnya through the oral transmission of information and from the examples set by the behavior of the older generation, formed an understanding, even if sometimes vague, of what Chechen cultural behavior should be based on. This included knowing Chechen language, observing religious rites, following cultural norms of etiquette, and being attentive to the dress-code and traditional gender roles. As the author's fieldwork conducted in 2014–2017 confirms, this is a general view of the first-generation migrants on what Chechen identity is or should be like (Iliyasov, 2018). The normative “should” used in the sentence before implies that it was not always the case that all the norms of cultural etiquette or religion were observed. This, as further presented cases highlight, obviously had an impact on the identity formation of the second-generation Chechens in Europe.

## The Identity Choices of Chechen Youth

### Muslim^8^

Muslim (the name)^9^ does not remember when or how he arrived in Europe. He was just a toddler, a little bit older than 2-years-old. He was the only child at that time, but his family grew shortly after. Now he is in his late teens and has a younger sister and brother. Muslim always felt responsible for his younger siblings because he is the eldest in the family, and he is a man. He is also especially protective of his sister. He always accompanies her when she needs to go out to ensure that she is safe and that she does not engage in any “unauthorized” communication. He has very strict views regarding his sister - he does not want her to see anybody before marriage.

Of course, she might get to know somebody through our friends or the Internet. They can communicate for a while and then marry. It is not that she should see him often before the marriage. This is how it should be. We are Chechens and we should follow our way. Our girls should marry only Chechen guys and our guys should marry only Chechen girls. If there is no candidate, she should stay single. Religion allows her to marry a Muslim of another ethnicity, but I would prefer her not to. They are different, even if they are our brothers. They do not understand how they should behave in front of the older people, when a young person can speak and when not, what vocabulary to use when you speak to the elderly and what is not allowed even with peers, what is good and what is bad (Interview with Muslim, June 2020).

The youngster has very rigid ideas regarding ethnic endogamy. He is particularly against the mixed marriages of Chechen females but has a less strict attitude toward the Chechen males' choices. Even though Muslim lives in a liberal society, he is not in favor of granting liberty to Chechen females. The young man is a religious person. He observes all prescriptions of Islam, but it does not bring him closer to other Muslims when it comes to mixed marriages and the choices of his sister. Despite his strict views, he has friends from different ethnic groups. Some of them are his schoolmates and others are from his sport's club where he would regularly train because he believes that a male should be strong and able to defend himself and his family.

I would prefer to hang out with Chechens. Unfortunately, there are not many Chechens around. I have some Chechen friends and we see each other occasionally. Now, with the pandemic (Covid-19), it became nearly impossible. I do not even go to play sports, let alone see my Chechen friends. Usually, we would meet when there was an occasion. My father has some Chechen friends and our families gather sometimes, but it is not very often. On some occasions, I meet new Chechens, when my father is invited to somebody's birthday or a wedding. In these festivities, we have good opportunities to meet other people, to communicate, to spend some time together, and to learn something about Chechnya from older people. Unfortunately, it is very rare and with Covid, it has become nearly impossible. Sometimes I communicate with my Chechen mates through WhatsApp, but this is not the same as live communication (Interview with Muslim, June 2020).

Muslim has a clear inclination toward practicing ethnic friendship. However, reality does not allow for this. Therefore, he has close friends from different ethnic groups. He likes them but they are not what he would want them to be. On the other hand, there is a question regarding whether his “ideal” friends would not disenchant him if they communicated daily. His views and attitudes are derived from his family education. Muslim's family is very traditional. His father has set up many rules at home. They do not watch Russian TV, they speak only Chechen at home (although this rule is constantly violated by both the parents themselves and the children alike), they show due respect to their guests and others around, and they observe all the regulations of Islam. As his father explains, they are Muslims of a Chechen origin, and he wants it to continue this way.

At a certain point, I was afraid that he will not become “boersh” (a Chechen word that describes manly behavior or manhood). To my understanding, he was too weak…, he had liberal views, even if he was doing sports and had a strong body, he did not have the right mindset. Once, there was a situation. We were sitting in the car and waiting for my daughter to come. There were some young drunk guys and… the situation developed. I told him: “Look, what in such situations a man should do.” I got out of the car and got myself involved. I almost went to prison for this later, but I gave him an example, and then I saw that he learnt a lesson from this. Now, I am calm. I taught him how to be a Chechen and I am proud of it. I was always patriotic in this sense. I agree that we are Muslims first, but it does not mean that we must abandon our Chechenness for this. Every Muslim nation is different, and many Arabs try to “sell” their behavior as Islam-based, which it is not. Therefore, to emphasize that I am Chechen, I have always worn a Chechen sheepskin hat, even when I was mingling with Salafists in Chechnya (Interview with Muslim's father, June 2020).

This excerpt from a conversation with Muslim's father sheds some light on the young man's behavior and attitudes as well as his friendship and marriage preferences. His parents put a lot of effort in to instill him with nationalism/patriotism. Although the young man has never visited his homeland and hardly knows what kind of life society lives there, he favors the in-group, which for him is limited by the Chechen migrants. He has no desire to explore his roots or contribute to the well-being of Chechnya, which he considers to be an occupied territory due to his father's instruction. Instead, Muslim would want to support Chechen communities in Europe. His communication with his homeland is limited. He shows up in front of his father's computer screen when requested to communicate with his relatives. However, Muslim does not show a real interest in keeping in touch with his extended family, even if this initiative, just as many others are, is supported by his mother too. These efforts, as she claims, consume all her energy.

There is nobody to help – no grandparents, aunties, no Chechen surrounding that my children could learn from. Therefore, it is all on us. He (it is not in a Chechen tradition to pronounce the name of husband) spends all his free time with them. He taught them a lot about our culture, our history, and traditions. We are lucky that he knows it all. In my turn, I always teach my girl to cook, explain to her what she should wear and why. I taught her to take care of her brothers because when she gets married, she will have to take care of her husband and his brothers (Interview with Muslim's mother, June 2020).

In sum, the case of Muslim demonstrates a very successful example of the transmission of original Chechen identity, as it is imagined by Muslim's parents and many other Chechen migrants (Iliyasov, 2018). They consider themselves being traditional Chechens and they are determined to raise their children the same way that their parents raised them. The dominance of the different culture had its imprint during the process of education – the first language of all their children is neither Chechen nor Russian. However, as the father of Muslim argued, his children are ready “to go to Chechnya right now and live there if necessary.” This means that they can protect themselves and adapt to the harsh conditions in which Chechen society lives. More than that, they have assumed their gender roles and learned Chechen behavior. As for the language, according to Muslim's father, it can be learned later.

### Abubakar^10^

The family of Abubakar arrived in Europe in 2003. Abubakar was in his pre-primary-school years at the time and he does not remember what hardships his family underwent moving from Chechnya. He knows it though. He also knows the reasons why they moved out from Chechnya and he has very clear political views regarding the matters inside the republic and around it. He monitors the situation there through online media and he is subscribed to some opposition social media channels which he receives updates and other historical materials from.

According to the local standards, the family of Abubakar is large. All in all, there are five children, and he is in the middle. The two elder sisters are married and live separately, which makes him the eldest. Abubakar has two younger sisters and grandparents who live next door. Every day he spends some time with his grandfather. It seems that they are best friends. Abubakar has other friends too, but it does not seem that he meets them daily. The grandfather, who was a schoolteacher and taught Chechen language at school, is delighted to spend time with his grandson. He sees it as his mission to pass all the knowledge that he has accumulated in his life to Abubakar. The latter enjoys it a lot. He listens to stories about their ancestors and about the Chechen heroes and religious figures. Abubakar has a lot of questions, which never remain unanswered.

I do not know, what would I be without my grandpa. He knows so much, and he is so interesting. My father would never manage to give me so much, he is always busy with work, and after work, he is too tired to spend time with me, and then I am busy with my stuff too. I must study a lot and do not have much free time. I would never learn about Chechnya and my ancestors. It happens that my college classmates ask me about Chechnya, and I would have nothing to say if not for my grandpa. I do not hang out much with my peers. Not many of my Chechen peers are interested in studying. They are more into sports and if not the career in UFC (Ultimate Fighting Championship) they dream about the jobs in security. Some already got into trouble with criminals. I think we (Chechens) should be especially law-obedient here, in the country that hosts us, and we should be useful for the society not only as “torpedoes” (Criminal slang used to describe men for action) (Interview with Abubakar, July 2017).

Despite only being in his late teens, he speaks maturely. He appreciates the efforts of his grandfather and considers him essential for his knowledge about Chechnya and Chechens. In other words, it is his grandfather who contributed the most to the construction of his ethnic and religious identity. Abubakar speaks perfect Chechen without any foreign accent. Moreover, he can easily write and read in Chechen, which is very rare, especially among children of Chechen migrants. These achievements are not surprising, given that the grandfather, who has a lot of free time, designed a serious education program for his grandson.

My grandchildren know everything about Chechnya and their ancestry. They are proud of it. We come from the very ancient line and we had statesmen and religious leaders among our ancestors. I explained to the kids that we [the Chechens] always wanted to be free and that Russia always wanted to enslave us. I explained to them the political interest of other nations too. They know that we were always betrayed by our so-called “brothers in Islam.” It was their fault behind the second war [of 1999–2009], they tried to use us to achieve their own goals and we were stupid to follow them. I am proud that my grandchildren know it and that they try to receive the best possible education here in Europe. I told them that it is the only way that we [Chechens] can achieve something. Now they are good citizens, living in a free country, and at the same time, they are patriots of Chechnya. They are ready to go there and work for the Chechen people (Interview with Abubakar's grandfather, July 2017).

The grandfather of Abubakar is proud of the results of his efforts. He believes that it was his education that made his grandchildren good citizens in their new home and patriots of Chechnya. He spoke to them about Chechen traditions, etiquette, and religion. He also explained the subtleties and differences of the branches of Islam, politics, and history. The former teacher at school was fully engaged with the private education of his grandchildren to whom he taught everything he thought was essential to know about Chechnya. He also encouraged them to benefit from the local system of education and to receive the best possible education. Education, in his mind, is the key to Chechnya's future, and he sees his grandchildren working for the Chechen people 1 day, being useful to them. Therefore, he has prepared them to return, which he considers necessary once the current regime is wiped out, something he is sure will happen soon. His discourse is ethnic centered. Although he considers religion to be the foundation of everything, he is mostly focused on his grandchildren fostering their ethnic identity. He taught them to be respectful toward others, particularly the elderly, and he taught them that they should prioritize Chechens over other Muslims. Even if he claims differently, he ensured that their primary identity is ethnic, and that religious identity is secondary.

Abubakar's parents Ali and Makka are happy that their children have grandparents nearby. According to Ali, the family would not be complete without them. Makka supports his view and is very grateful for all the help that she receives from her mother-in-law, while she is at work.

We would have never succeeded to raise children as Chechens here. It is just impossible. We are all the time at work and after work, we are too tired to do something with our children. But Ali's parents are free, they are pensioners and they are still very active. So, they dedicated all free time to helping us with the children. I can't even imagine what would it be without them. I do not want my children to spend much time with other Chechen youth outside. Many are too naughty, and I am afraid that my children would pick up something bad from them. Non-Chechen youth is even worse, they have very disrespectful behavior and I would be devastated if my children were like them (Interview with Abubakar's mother, July 2017).

To sum up, the case of Abubakar illustrates one more successful example of preserving his parents original ethnic identity. The identity of this youngster is probably closest to the one of his father, as it was transmitted to him by his grandparents – the same people who raised Ali. The home environment and parents'/grandparents' negative attitude regarding communication with the children's peers protected them from being influenced by the host society and other migrant groups, which contributed to the ethnic identity growing stronger.

### Deni^8^

Deni likes his name. He calls it “convertible,” like a car. Whenever it is needed, he can be Danny, Danis, Denis, etc. This universality of his name gives him options to wear different “masks” as he called his various identities. His “Chechen mask” he puts on with his family, so as not to upset his mother. Even if he knows only a few basic phrases in Chechen and does not stand up to greet the elders, he shows a certain respect to his parents. With this he keeps a bond, even if only symbolic, with his Chechenness. Other “masks” are mostly for communication with his peers and his social image. Usually, he is Danny. Deni plays guitar in a small band. He stays day and night in the garage of one of his bandmates. Sometimes, he visits home to change his clothes and to eat. He can sleep a night or two at home (in a week), but mostly he prefers to spend his time with his band and his girlfriend. He works part-time in the local petrol station and this gives him enough money to eat McDonald's or buy some extra clothes. Sometimes, he even contributes to the family budget.

I do not communicate with my Chechen peers. They are very judgmental. First, I had some Chechen friends, but then I decided to stay away from them. They were older than me and they pressured me a lot to behave as they considered it was correct. I did not feel free with them. I want to have long hair, I want to wear what I want, I want to do what I want. After all, we live in a free society, why should we do something that we do not want? It upsets my parents a little bit that I do not have Chechen friends, but I do not do anything bad. I am not a murderer, I am not a drug dealer, so why can't I be free and do what I want? I want to become a famous musician and it is my dream to be successful in this field. I know that I am a Chechen by birth, so what? Is it something that people should be judged by? By being French, German, or English? I don't think so (Interview with Deni, June 2020).

Deni does not care about his membership in his ethnic group. It is too demanding for him. Being a Chechen requires observing traditions, not wearing certain clothes, and having the “correct” haircut. Therefore, he prefers to befriend members of his host society or Russians – another migrant group that he shares a common language with. The only bond that keeps him with his ethnicity is his direct family. He is the only child and his parents try to pacify with his choices in order not to lose him completely. He barely speaks with his father, and all his communication, as is quite conventional in Chechen families, goes indirectly – through mother. They are not on bad terms, but Deni knows that father does not approve of his choices, something which distances them. His father Arbi also knows Deni's position.

I do not want to pressure him, but the problem is that whatever I say, he takes it wrong, as if we are enemies. So, I decided just to comply with whatever happens. I am not very religious myself and I never forced him to pray. I do not like very religious people; I think many of them are hypocrites. You cannot say it about everyone, but many are for sure. I often witnessed how people, who pretend to be very religious, do things that neither go along with religion nor with the understanding of the normal people. At the same time, you can see that many people here are not religious at all and, despite this, they behave in a way that makes them more humane than those who claim to be religious. I guess, this hypocrisy of so-called religious people turned me off religion. But it is not the only reason why Deni is like this. I never forced him to keep in touch with our relatives in Chechnya, and I never forced him to befriend Chechen guys. He always had his own choices. I don't know, maybe I was wrong and maybe I should have beat all the bad out of him, but I do not believe in this method of education. So, it is as it is, I only hope that he will become a normal human being. I do not think he will ever return to Chechnya, and probably I won't either. We did not visit our relatives for several years, because it took time before we received our passports, and when we did, there was not even a question of him going with us. I knew he preferred to stay here. We don't have many Chechens around us either. Therefore, we do not get to communicate often, even if I would like to (Interview with Deni's father, June 2020).

Arbi's discourse is very explicit. He names several reasons why Deni has chosen to drift away (at least for now) from the image of what Chechen identity/behavior should be. Among them is his own decision to integrate fully into the host society. Other than that, he and his wife did not invest much time in instilling Chechen norms of cultural etiquette or religious behavior in the boy as they do not observe all of them either. The reason for this is that they do not have a “Chechen surrounding.” To add to this, before coming to Europe, the family spent several years in Russia, where the boy was attending kindergarten and primary school. Therefore, he speaks perfect Russian (and the local language), but he does not speak much Chechen. He understands the basic phrases, but since his first language is Russian, his parents were forced to switch to this language too. They do not communicate in Chechen much even amongst themselves. There is a sound of regret in the voice of Arbi when he speaks about his son and his “non-Chechen” behavior, but he comforts himself by saying that it is more important to be a good person rather than to be a Chechen or a Muslim.

In sum, the case of Deni illustrates an outcome different from the two previously analyzed cases, which demonstrated quite a successful transmission of the original Chechen identity as it is imagined by the first-generation Chechen migrants. Deni was raised in a non-original society and had the example of his parents, who have prioritized integration into the host society pratly sacrificing their original identity. In other words, the original identity that the boy received from his parents was already weakened. His parents recognize it and even slightly regret blaming the disadvantageous circumstances for the boy's subsequent drifting away from the image of a Chechen that they once had in mind.

## Conclusion

This paper aimed at identifying the factors that play a crucial role in identity formation of second-generation Chechens in Europe. To achieve this goal, the article outlined the original identity of first-generation migrants and identified the three main factors that mostly impact the identity formation of migrants' children. These factors (family, host society, and other migrant groups within host society) were classified as main, primary, secondary, and tertiary. All these factors are related to the social milieu that migrants and their children live in daily. They also influence the identity construction of migrants the most. This is not to diminish the importance of the personal characteristics of the separate individuals, the analysis of which would necessitate a separate investigation.

The study was limited by only three analyzed cases, which do not collectively represent all the possible choices or outcomes. Having in mind a huge amount of possible individual outcomes of identity formation and negotiation process (Kość-ryzko, 2015), the article refuses to claim generalisability of the studied cases. Furthermore, the article acknowledges gender-based limitation of this study and suggests researching this aspect separately.

Basing the analysis on the developed theoretical framework, the article considers three different social surroundings that could have influenced migrant children's collective identity formation in each of the chosen cases. In all three cases, the investigation confirmed Rabiau (2019) conclusion that the most important surrounding of all was the family one. The efforts of the parents and input in home education influenced the outcome of identity formation of the Chechen youth. So did the atmosphere in the house as well as the parents' attitude toward their own collective identity (see also McGregor et al., 2015, 2016). These factors, as the study demonstrates, determined the formation of the youth's worldview, interest in the original identity of their parent, their decision to be a Chechen or not, and their communication preferences.

Further, this research partly confirmed the findings established by the earlier studies (Druckman, 1994; Lee, 1999; Luo and Wiseman, 2000), which concluded that the influence of the host society was proportionate to the size of the migrant's community. However, the possibility to communicate with peers from the same ethnic group was not the decisive factor, though it was an important one, in the choices of the analyzed youth. This study also found that the influence of other migrant communities was much less intense than the factors of family or home education and the dominance of the host society. The impact of the latter was mostly obvious in terms of language preference and knowledge of the cultural and historical basics.

In addition to this, the analyzed cases illustrated the width of the choices that second-generation Chechens in Europe can and do have. This width stretches from, but is not limited to, the almost preserved (rather successfully transmitted) original identity, as it is imagined by the first-generation Chechen migrants, to a significant drift-away from the model. As the cases illustrate, the attachment to collective identity varies within the group. The external expressions of this attachment in Chechen migrant communities are demonstrated through the strict observation (or absence of it) of the ethnic rites, through participating (or not) in folk celebrations, through practice (or not) of the ethnic endogamy, through the aspiration (or lack of it) to transmit original identity to their children, through rallying (or not) behind the flag of the homeland, and through knowledge (or lack of it) of the nation's history.

## Data Availability Statement

The raw data supporting the conclusions of this article will be made available by the authors, without undue reservation.

## Ethics Statement

The studies involving human participants were reviewed and approved by University of St Andrews, School of International Relations. The patients/participants provided their written informed consent to participate in this study. Written informed consent was obtained from the individual(s) for the publication of any potentially identifiable images or data included in this article.

## Author Contributions

The author confirms being the sole contributor of this work and has approved it for publication.

## Conflict of Interest

The author declares that the research was conducted in the absence of any commercial or financial relationships that could be construed as a potential conflict of interest.

## Footnotes

^1^By “original” the paper means the identity that migrants had formed while they lived in Chechnya before moving to Europe.

^2^Vstrecha s Khristom v Ramadan 05/07/2020. Available online at: https://www.youtube.com/watch?v=XAxv1iNOsaU (accessed October 6, 2020).

^3^Ona ne chechenka 20/02/2020. Available online at: https://www.youtube.com/watch?v=H527lS1lhZo (accessed October 6, 2020).

^4^This study is limited to the exploration of Chechen teenage-male identity formation leaving the question of possible influence of discussed identity-forming factors on teenage-female for further research.

^5^Ethnic groups in the Chechen and Ingush Soviet Socialist Autonomous Republic, according to the 1979 census. Available online at: http://www.demoscope.ru/weekly/ssp/rus_nac_79.php?reg=51 (accessed October 15, 2020). Ethnic groups in the Chechen and Ingush Soviet Socialist Autonomous Republic, according to the 1999 census. Available online at: http://www.demoscope.ru/weekly/ssp/rus_nac_89.php?reg=49 (accessed October 15, 2020).

^6^Personal interview of a Chechen male, 56 years old. Belgium, March 2017.

^7^Personal interview of a Chechen male, 47 years old. Belgium, March 2017.

^8^Fieldwork of 2020 (Muslim's and Deni's cases).

^9^All names are changed to protect the identity of the informants.

^10^Fieldwork of 2017 (Abubakar's case).

## References

[B1] Abdelal R. Herrera Y. M. Johnston A. I. McDermott R. (eds.). (2009). Measuring Identity: A Guide for Social Scientists. Cambridge: Cambridge University Press. 10.1017/CBO9780511810909

[B2] Ackroyd J. Pilkington A. (1999). Childhood and the construction of ethnic identities in a global age: a dramatic encounter. Childhood 6, 443–454. 10.1177/0907568299006004004

[B3] Adams G. R. Marshall S. K. (1996). A developmental social psychology of identity: understanding the person-in-context. J. Adolesc. 19, 429–442. 10.1006/jado.1996.0041 9245296

[B4] Albert C. D. (2014). The ethno-violence nexus: measuring ethnic group identity in Chechnya. East European Politics 30, 123–146. 10.1080/21599165.2013.848796

[B5] Baumeister R. F. (1995). Self and identity: an introduction. Adv. Soc. Psychol. 93, 51–97.

[B6] Berry J. W. (2001). A psychology of immigration. J. Soc. Iss. 57, 615–631. 10.1111/0022-4537.00231

[B7] Booth W. J. (1999). Communities of memory: on identity, memory, and debt. Am. Pol. Sci. Rev. 93, 249–263. 10.2307/2585394

[B8] Bosma H. A. Kunnen E. S. (2001). Determinants and mechanisms in ego identity development: a review and synthesis. Dev. Rev. 21, 39–66. 10.1006/drev.2000.0514

[B9] Burke P. J. (2006). Identity change. Soc. Psychol. Q. 69, 81–96. 10.1177/019027250606900106

[B10] Crocetti E. Fermani A. Pojaghi B. Meeus W. (2011). Identity formation in adolescents from Italian, mixed, and migrant families. Child Youth Care Forum 40, 7–23. 10.1007/s10566-010-9112-8

[B11] Crocetti E. Rubini M. Meeus W. (2008). Capturing the dynamics of identity formation in various ethnic groups: development and validation of a three-dimensional model. J. Adolesc. 31, 207–222. 10.1016/j.adolescence.2007.09.002 17959238

[B12] De Castro L. R. (2004). Otherness in me, otherness in others: children's and youth's constructions of self and other. Childhood 11, 469–493. 10.1177/0907568204047107

[B13] Devine D. Kelly M. (2006). ‘I just don't want to get picked on by anybody': dynamics of inclusion and exclusion in a newly multi-ethnic Irish primary school. Children Society 20, 128–139. 10.1111/j.1099-0860.2006.00020.x

[B14] Druckman D. (1994). Determinants of compromising behavior in negotiation: a meta-analysis. J. Conflict Resolut. 38, 507–556. 10.1177/0022002794038003007

[B15] Eriksen T. H. (1993). Formal and informal nationalism. Ethn. Racial Stud. 16, 1–25. 10.1080/01419870.1993.9993770

[B16] Gammer M. (2006). The Lone Wolf and the Bear. Pittsburgh, PA: Univ. of Pittsburgh press.

[B17] Guzder J. (2011). Second skins: family therapy agendas of migration, identity, and cultural change. Fokus på familien 39, 160–179.

[B18] Hughes D. Johnson D. (2001). Correlates in children's experiences of parents' racial socialization behaviors. J. Marr. Family 63, 981–995. 10.1111/j.1741-3737.2001.00981.x

[B19] Idema H. Phalet K. (2007). Transmission of gender-role values in Turkish-German migrant families: the role of gender, intergenerational and intercultural relations. Zeitschrift für Familienforschung 19, 71–105.

[B20] Iliyasov M. (2018). Chechen ethnic identity: assessing the shift from resistance to submission. Middle East. Stud. 54, 475–493. 10.1080/00263206.2018.1423967

[B21] Jaimoukha A. (2004). The Chechens: A Handbook. Abingdon: Routledge. 10.4324/9780203356432

[B22] Kibria N. (2002). “Of blood, belonging, and homeland trips: transnationalism and identity among second-generation Chinese and Korean Americans,” in The Changing Face of Home: The Transnational Lives of the Second Generation, eds P. Levitt and M. Waters (New York, NY: Russell Sage Foundation), 295–311.

[B23] Kinnvall C. (2004). “Globalization, identity, and the search for chosen traumas,” in The Future of Identity: Centennial Reflections on the Legacy of Erik Erikson, ed K. Hoover (Lanham MD: Lexington Books), 111–136.

[B24] Kirilenko A. (2017). Chechen Refugees in Europe: Reasons Why They Flee Russia and Asylum Problems. Available online at: https://legal-dialogue.org/chechen-refugees-europe-reasons-flee-russia-asylum-problems (accessed October 16, 2020).

[B25] Kość-ryzko K. (2015). “Identity formation of young chechen refugees in poland in the face of foreign cultures,” in Contextualizing Changes: Migrations, Shifting Borders and New Identities in Eastern Europe, eds P. Hristov, A. Kassabova, E. Troeva, and D. Demski (Sofia: Paradigma Ltd), 315–329.

[B26] Kosic A. Mannetti M. Sam D. L. (2005). The role of majority attitudes towards out-group in the perception of the acculturation strategies of immigrants. Int. J. Intercult. Relat. 29, 273–288. 10.1016/j.ijintrel.2005.06.004

[B27] Laruelle M. (2017). Kadyrovism: Hardline Islam as a Tool of the Kremlin? Paris Cedex 15: IFRI.

[B28] Lee S. J. (1999). “Are you Chinese or What?” Ethnic Identity Among Asian Americans (Mahwah, NJ: Lawrence Erlbaum Associates Publishers).

[B29] Luo S. H. Wiseman R. L. (2000). Ethnic language maintenance among Chinese immigrant children in the United States. Int. J. Intercult. Relat. 24, 307–324. 10.1016/S0147-1767(00)00003-1

[B30] Mach Z. (1993). Symbols, Conflict, and Identity: Essays in Political Anthropology. Albany, NY: SUNY Press.

[B31] Maleševic S. (2003). Researching social and ethnic identity: a skeptical view Sinisa Maleševic'. J. Language Polit. 2, 265–287. 10.1075/jlp.2.2.05mal

[B32] McBride Murry V. Brown P. A. Brody G. H. Cutrona C. E. Simons R. L. (2001). Racial discrimination as a moderator of the links among stress, maternal psychological functioning, and family relationships. J. Marriage Family 63:915. 10.1111/j.1741-3737.2001.00915.x

[B33] McGregor L. S. Melvin G. A. Newman L. K. (2015). Familial separations, coping styles, and PTSD symptomatology in resettled refugee youth. J. Nerv. Ment. Dis. 203, 431–438. 10.1097/NMD.0000000000000312 25993333

[B34] McGregor L. S. Melvin G. A. Newman L. K. (2016). An exploration of the adaptation and development after persecution and trauma (ADAPT) model with resettled refugee adolescents in Australia: a qualitative study. Transcult. Psychiatry 53, 347–367. 10.1177/1363461516649546 27207591

[B35] Molodikova I. (2019). “Muslim refugees from russia: do the chechens bring their own ‘aul' from chechnya to the EU?” in Muslim Minorities and the Refugee Crisis in Europe, eds K. Górak-Sosnowska, M. Pachocka, and J. Misiuna (Warsaw: SGH Publishing House), 119–133.

[B36] Parham T. A. Helms J. E. (1981). The influence of Black students' racial identity attitudes on preferences for counselor's race. J. Couns. Psychol. 28:250. 10.1037/0022-0167.28.3.250

[B37] Patackas A. (1999). “Kodel Cečenija?” [Why Chechenia?]. Naujasis Židinys, no. 11-12.

[B38] Paul M. J. Fischer J. L. (1980). Correlates of self-concept among Black early adolescents. J. Youth Adolesc. 9, 163–173. 10.1007/BF02087934 24318018

[B39] Phalet K. Schönpflug U. (2001). Intergenerational transmission of collectivism and achievement values in two acculturation contexts: the case of Turkish families in Germany and Turkish and Moroccan families in the Netherlands. J. Cross Cult. Psychol. 32, 186–201. 10.1177/0022022101032002006

[B40] Phinney J. S. (2000). Identity formation across cultures. Hum. Dev. 43, 27–31. 10.1159/000022653

[B41] Phinney J. S. Chavira V. (1992). Ethnic identity and self-esteem: an exploratory longitudinal study. J. Adolesc. 15, 271–281. 10.1016/0140-1971(92)90030-9 1447413

[B42] Rabiau M. A. (2019). Culture, migration, and identity formation in adolescent refugees: a family perspective. J. Fam. Soc. Work 22, 83–100. 10.1080/10522158.2019.1546950

[B43] Rousseau C. Mekki-Berrada A. Moreau S. (2001). Trauma and extended separation from family among Latin American and African refugees in Montreal. Psychiatry 64, 40–59. 10.1521/psyc.64.1.40.18238 11383441

[B44] Ryan L. (2010). Becoming Polish in London: negotiating ethnicity through migration. Soc. Identities 16, 359–376. 10.1080/13504630.2010.482425

[B45] Sigel R. (ed.). (1989). Political Learning in Adulthood. Chicago: University of Chicago Press.

[B46] Sipos M. (2020). “We are all brothers here”: the making of a life by Chechen refugees in Poland. Popul. Space Place 26:2276. 10.1002/psp.2276

[B47] Sleijpen M. Boeije H. R. Kleber R. J. Mooren T. (2016). Between power and powerlessness: a meta-ethnography of sources of resilience in young refugees. Ethnicity Health 21, 158–180. 10.1080/13557858.2015.1044946 26107385

[B48] Sokirianskaia E. (2005). Families and clans in Ingushetia and Chechnya. A fieldwork report. Central Asian Surv. 24, 453–467. 10.1080/02634930500453590

[B49] Szczepanikova A. (2012). Becoming more conservative? Contrasting gender practices of two generations of Chechen women in Europe. Eur. J. Women's Stud. 19, 475–489. 10.1177/1350506812466611

[B50] Szczepanikova A. (2015). Chechen women in war and exile: changing gender roles in the context of violence. Natl. Pap. 43, 753–770. 10.1080/00905992.2014.999315

[B51] Tajfel H. Turner J. C. (1986). “An integrative theory of group conflict,” in The Social Psychology of Intergroup Relations, eds W. G. Austin and S. Worchel (Brooks/Cole Publishing Company), 7–24.

[B52] Tishkov V. (2004). Chechnya: Life in a War-Torn Society, Vol. 6. Berkeley, CA: University of California Press. 10.1525/california/9780520238879.001.0001

[B53] Triandafyllidou A. (2009). Migrants and ethnic minorities in post-Communist Europe: negotiating diasporic identity. Ethnicities 9, 226–245. 10.1177/1468796809103461

[B54] Triandis H. C. (2001). Individualism-collectivism and personality. J. Pers. 69, 907–924. 10.1111/1467-6494.696169 11767823

[B55] Verkuyten M. Kinket B. (2000). Social distances in a multi-ethnic society: the ethnic hierarchy among Dutch preadolescents. Soc. Psychol. Q. 63, 75–85. 10.2307/2695882

[B56] Williams B. G. (2000). Commemorating “the deportation” in post-Soviet Chechnya: the role of memorialization and collective memory in the 1994-1996 and 1999-2000 Russo-Chechen Wars. History Memory 12, 101–134. 10.1353/ham.2000.0006

[B57] Williams B. G. (2016). Inferno in Chechnya: The Russian-Chechen Wars, the Al Qaeda Myth, and the Boston Marathon Bombings. Lebanon, NH: University Press of New England.

